# Predictors of residual tricuspid regurgitation after interventional therapy: an automated deep-learning CT analysis

**DOI:** 10.1038/s41598-024-70768-x

**Published:** 2024-08-27

**Authors:** Isabel Mattig, Elena Romero Dorta, Katherine Fitch, Alexander Lembcke, Marc Dewey, Karl Stangl, Henryk Dreger

**Affiliations:** 1 Department of Cardiology, Angiology and Intensive Care Medicine, Deutsches Herzzentrum der Charité, Campus Charité Mitte, Berlin, Germany; 2 Charité – Universitätsmedizin Berlin, Corporate Member of Freie Universität Berlin and Humboldt-Universität zu Berlin, Charitéplatz 1, 10117 Berlin, Germany; 3 DZHK (German Centre for Cardiovascular Research), Partner Site, Berlin, Germany; 4 Berlin Institute of Health at Charité – Universitätsmedizin Berlin, BIH Biomedical Innovation Academy, Berlin, Germany; 5 Laralab GmbH, Munich, Germany; 6 Department of Radiology, Charité – Universitätsmedizin Berlin, Corporate Member of Freie Universität Berlin and Humboldt-Universität zu Berlin, Charitéplatz 1, 10117 Berlin, Germany; 7 Berlin University Alliance, BAU, Berlin, Germany; 8 Department of Cardiology, Angiology and Intensive Care Medicine, Deutsches Herzzentrum der Charité, Campus Virchow Klinikum, Augustenburger Platz 1, 13353 Berlin, Germany; 9 Structural Heart Intervention Program (SHIP), Deutsches Herzzentrum der Charité, Berlin, Germany

**Keywords:** Cardiology, Interventional cardiology

## Abstract

Computed tomography (CT) is used as a valuable tool for device selection for interventional therapy in tricuspid regurgitation (TR). We aimed to evaluate predictors of TR reduction using CT and automated deep learning algorithms. Patients with severe to torrential TR and CTs prior to either percutaneous annuloplasty (PA) or tricuspid transcatheter edge-to-edge repair (T-TEER) were enrolled. CTs were analyzed using automated deep learning algorithms to assess tricuspid valve anatomy, right heart morphology, and function. Outcome parameters comprised post-interventional TR ≤ 1 and all-cause mortality. 84 patients with T-TEER (*n* = 32) or PA treatment (*n* = 52) were enrolled. Patients with a post-interventional TR ≤ 1 presented lower tenting heights and smaller tenting angles compared to patients with a TR > 1. Tenting height showed the best accuracy for post-interventional TR > 1 with an AUC of 0.756 (95% CI 0.560–0.951) in the T-TEER and 0.658 (95% CI 0.501–0.815) in the PA group, consistent with a suggested threshold of 6.8 mm and 9.2 mm, respectively. Patients with a post-interventional TR ≤ 1 exhibited a mortality of 4% and those with a TR > 1 of 12% during a follow-up of 331 ± 300 and 370 ± 265 days, respectively (*p* = 0.124). To conclude, tenting is associated with procedural outcomes and should be considered during screening for interventional TR therapy.

## Introduction

Tricuspid regurgitation (TR) has a significant impact on survival leading to a relevant increase in mortality^1^. Particularly in the elderly, the prevalence of TR increases and results in a substantial disease burden^2^. Surgical repair or replacement is effective in TR reduction but also associated with a high intra-hospital mortality rate up to 10%^3^. Consequently, there is a need for new treatment options besides surgical and symptomatic medical therapy. In recent years, new interventional therapies were developed for patients with a high operative risk and demonstrated a significant impact on TR reduction and improvement of functional capacity^4–9^. Percutaneous annuloplasty (PA) and tricuspid transcatheter edge-to-edge repair (T-TEER) are the most used approaches and several algorithms for device selection have been proposed^4,8,9^. However, predictors of post-interventional TR reduction remain limited.

Procedural planning is essential for successful interventional therapy. While echocardiography is the primary modality for TR assessment, computed tomography (CT) serves as an alternative method to measure tricuspid valve anatomy, right heart morphology, and function^10^. CT assessment is used to evaluate tricuspid annular dimensions, location of leads from cardiac implantable electronic devices (CIEDs) and the proximity of the tricuspid annulus to the right coronary artery (RCA)^8^. Moreover, cardiac CT is necessary to guide the optimal placement of anchor points in patients undergoing PA^8^. In addition to procedural planning, parameters assessed by cardiac CT can predict post-interventional outcome^11^. Tanaka et al. observed an association between a reduced right ventricular ejection fraction (RVEF) measured by CT and an increase in all-cause mortality and hospitalization due to heart failure 1 year after interventional therapy^11^.

Since CT is typically performed during TR evaluation and procedural planning of both PA and occasionally T-TEER, integrating its information into treatment algorithms is essential for optimizing patients’ therapy and device selection. Therefore, the focus of our current study was to assess tricuspid valve anatomy, right heart morphology, and function by CT using automated deep learning algorithms in patients who underwent PA or T-TEER. Furthermore, we aimed to evaluate parameters associated with residual TR after interventional therapy.

## Methods

### Study design

We enrolled patients who underwent PA (Cardioband®, Edwards Lifesciences, Irvine, CA, USA) or T-TEER (TriClip®, Abbott, Chicago, Illinois, USA, or PASCAL®, Edwards Lifesciences, Irvine, California, USA) from 2019 to 2023 due to symptomatic severe to torrential TR despite optimal medical therapy. Due to availability of automated CT analysis since 2022, we performed a retrospective analysis of previous cases and a prospective analysis of new patients (2019–2023). Additional inclusion criteria comprised a cardiac full cycle CT during the screening process prior to interventional therapy. Patients with combined procedures of the tricuspid and mitral valve and patients with a second TR procedure (e.g. PA following T-TEER) were excluded from the analysis.

All patients underwent TR evaluation prior to interventional therapy which includes echocardiographic examination, cardiac CT and right and left heart catheterization. The device selection and recommendation of our local heart team was based on the individual tricuspid anatomy and function^8,9,12^. In detail, PA was mainly chosen in patients with atrial TR, a central jet, and a sufficient distance from the hinge point to the right coronary artery^12^. In contrast, patients with ventricular TR, a more commissural jet, and a coaptation gap of less than 8 mm were treated with T-TEER^12^. The decision tree is published elsewhere^12^. If PA appeared more feasible or T-TEER was challenging or not feasible, patients underwent a complete work-up including a cardiac CT. Moreover, while some of the TR patients seemed to be good candidates for PA based on echocardiography, the CT scan revealed anatomical characteristics such as prohibitive RCA proximity. Accordingly, these patients were treated with T-TEER and CT scans were available for the present analysis. Since establishing a prospective registry, all patients were invited to receive follow-up examinations. Written informed consent was provided by patients with follow-up examinations; the need for written informed consent of patients with retrospective CT analysis was negated by our institutional review board and data protection service. The study was authorized by the institutional review board of the Charité – Universitätsmedizin Berlin, Germany (EA4/218/21 and EA1/043/20) and conducted in accordance with relevant guidelines and regulations, including the Declaration of Helsinki.

Echocardiography was performed in line with the standards of the American Society of Echocardiography (ASE) and the European Association of Cardiovascular Imaging (EACVI)^13,14^. The assessment comprised right ventricular morphology and function as well as TR quantification by effective regurgitant orifice area and regurgitant volume using the proximal isovelocity surface area method, vena contracta, and hepatic vein reflux^15^. According to current literature and recommendations, the leading cause of TR was categorized into primary, CIED-related, atrial or ventricular secondary TR and in case of more than one mechanism as mixed pathology^8,16–19^. A GE healthcare Vivid E9 or E95 with a M5S(c) 1.5–4.5 MHz or 4Vc-D 3D/4D Phased Array 1.4–5.2 MHz transducer (GE Vingmed, Horton, Norway) was used for echocardiography.

Technical success was defined as successful implantation of the device and retrieval of the delivery system without conversion to surgical or another interventional therapy as well as TR reduction of at least one grade. Outcome parameters comprised TR reduction and all-cause mortality during follow-up.

### Computed tomography

CT was performed before intervention during the screening process for TR therapy according to current recommendations^20,21^. For further analysis of the CTs, we used heart.ai version 1 (Laralab GmbH, Munich, Germany), a cloud-based software platform providing automatic analysis of cardiac CT images for research use (https://research.heartai-medical.com/). Heart.ai's computer modeling and simulation methods are based on deep learning techniques. Blood cavities and tissues of the heart were segmented using Convolutional Neural Networks, with different networks focusing on different groups of heart anatomies. The result was a comprehensive segmentation that included the major structures such as the left and right ventricles, as well as finer structures such as tricuspid leaflets. Segmentation was performed for each phase in a cardiac CT series, resulting in a detailed representation of the cardiac anatomy. 3D models of cardiac anatomy were produced from the binary masks of the segmentations using the flying edges algorithm^22^. Characteristic heart planes for multiplanar reconstruction views were calculated based on the 3D models, and custom algorithms were used to automatically derive a wide range of measurements, including annulus and right heart dimensions. The 3D models from a given CT series were collated to calculate phase-independent measurements, such as RV stroke volume. Furthermore, this allowed for 4D visualization of the heart's structural dynamics over the cardiac cycle. Automated cardiac CT analysis included the evaluation of right heart morphology and function, as well as TR measurements. Right atrial morphology was measured at the end-systolic phase, right ventricular morphology at end-diastolic phase, tricuspid annulus morphology at both end-systolic and mid-diastolic phases, and tenting parameters at the end-systolic phase. Manual adjustments were made if necessary. Tenting height as well as angles were measured by hand.

### Statistical analysis

We used SPSS Statistics version 28 for Windows (IBM Corporation, New York, NY, USA) for statistical analysis. Categorical and ordinal variables were presented in percentages and continuous variables as median with 25th and 75th percentile or mean with standard deviation (depending on their skewness, uniform per variable). Statistical tests for intergroup comparison comprised chi-squared test (categorical parameters), *t*-test (normally distributed, continuous parameters) and Wilcoxon-test or Mann–Whitney-U-test (not normally distributed continuous or ordinal parameters). Outcome predictors were assessed by univariate binary logistic regression analysis with the dependent parameter post-interventional TR grade ≤ 1. Moreover, receiver operating characteristic (ROC) analysis were conducted to calculate the accuracy of evaluated CT parameters. Correlation of CT and echocardiographic measurements were analysed using Pearson or Spearman's rank correlation, according to the value`s distribution. Survival analysis was performed using log-rank test and Kaplan–Meier curves. A *p* value < 0.05 was considered statistically significant.

### Ethics approval

The study was approved by the institutional review board of the Charité – Universitätsmedizin Berlin, Berlin, Germany (EA4/218/21 and EA1/043/20).

## Results

**Fig. 1:**
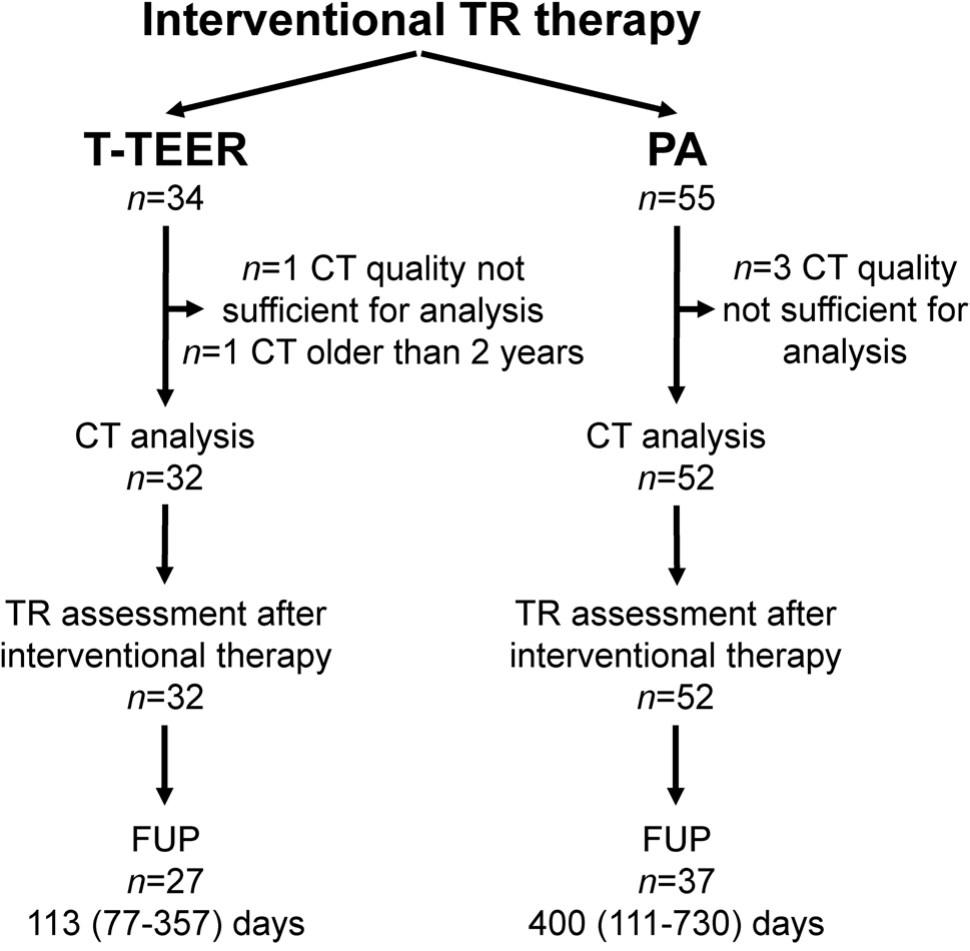
Flow chart of the study presenting the number of patients with tricuspid regurgitation (TR) who received tricuspid transcatheter edge-to-edge repair (T-TEER) or percutaneous annuloplasty (PA) and follow-up (FUP) examinations.

Between 2019 and 2023, 84 patients with a cardiac CT performed during the screening process were included in our analysis. CTs were conducted 64 (47–91) days prior to interventional therapy. Four patients with CTs not suitable for automated analysis and one patient with a CT performed 7 years before the procedure were excluded. Figure 1 presents the screening and enrolment process of the present study. Baseline characteristics are listed in Table 1. Overall, 32 patients underwent T-TEER and 52 patients PA. Most of the patients were female (63%) and had a median age of 81 (77–83) years. 88% of patients reported a NYHA class III or IV. There were no significant differences regarding baseline characteristics between both groups except for the prevalence of peripheral artery disease and the intake of Sodium-Glucose Transport Protein 2 (SGLT2) inhibitors.

**Table 1:** Baseline characteristics.

	T-TEER (*n* = 32)	PA (*n* = 52)	*p* Value
Female, *n* (%)	19 (59)	34 (65)	0.579
Age, years (IQR)	82 (78–84)	81 (77–83)	0.287
BMI, kg/m^2^ (IQR)	24 (22–27)	25 (22–28)	0.235
EUROScore II (IQR)	5.6 (3.5–8.7)	4.5 (3.0–8.4)	0.261
NYHA class			0.112
I, *n* (%)	0 (0)	0 (0)	
II, *n* (%)	2 (6)	8 (15)	
III, *n* (%)	25 (78)	40 (77)	
IV, *n* (%)	5 (16)	4 (8)	
Leading cause of TR			0.131
Primary TR, *n* (%)	1 (3)	1 (2)	
Atrial TR, *n* (%)	24 (75)	28 (54)	
Ventricular TR, *n* (%)	6 (19)	22 (42)	
CIED-related TR, *n* (%)	1 (3)	0 (0)	
Mixed pathology, *n* (%)	0 (0)	1 (2)	
Coronary artery disease, *n* (%)	12 (38)	30 (58)	0.072
CIED, *n* (%)	11 (34)	14 (27)	0.468
Atrial fibrillation or flutter, *n* (%)	28 (88)	50 (96)	0.135
Arterial hypertension, *n* (%)	26 (81)	45 (87)	0.515
Diabetes mellitus, *n* (%)	4 (13)	16 (31)	0.082
Stroke, *n* (%)	8 (25)	9 (17)	0.394
Peripheral artery disease, *n* (%)	9 (28)	2 (4)	0.001
Chronic obstructive pulmonary disease, *n* (%)	9 (28)	9 (17)	0.241
History of surgical or percutaneous valve therapy, *n* (%)	14 (44)	18 (35)	0.402
Serum creatinine, mg/dl (IQR)	1.3 (1.0–1.7)	1.3 (1.0–1.4)	0.652
NT-proBNP, ng/l (IQR)	1919 (1062–3775)	2463 (1711–4206)	0.191
Bilirubin, mg/dl (IQR)	1.0 (0.6–1.6), *n* = 26	0.6 (0.5–1.1), *n* = 33	0.145
Systolic PAP, mmHg ± SD	38 ± 14, *n* = 23	41 ± 13, *n* = 24	0.521
Heart failure therapy			
Diuretics, *n* (%)	31 (97)	51 (98)	0.726
Beta-blocker, *n* (%)	29 (91)	43 (83)	0.313
ACE inhibitor or sacubitril/valsartan, *n* (%)	22 (69)	42 (81)	0.209
Mineralocorticoid receptor antagonist, *n* (%)	20 (63)	31 (60)	0.793
SGLT2 inhibitor, *n* (%)	13 (41)	10 (19)	0.033

Continuous variables are shown as mean ± standard deviation (SD, normally distributed) or median with interquartile ranges (IQR, not normally distributed), categorical variables are given as absolute number with percentages. T-TEER, Tricuspid transcatheter edge-to-edge repair; PA, Percutaneous annuloplasty; BMI, Body mass index; EUROScore II; European System for Cardiac Operative Risk Evaluation II, NYHA class, New York Heart Association Class; TR, Tricuspid regurgitation; CIED, Cardiac implantable electronic device; NT-proBNP, N-Terminal pro brain natriuretic peptide; PAP, Pulmonary artery pressure; ACE, Angiotensin-converting enzyme; SGLT2, Sodium-Glucose Transport Protein 2.

### CT analysis

**Fig. 2:**
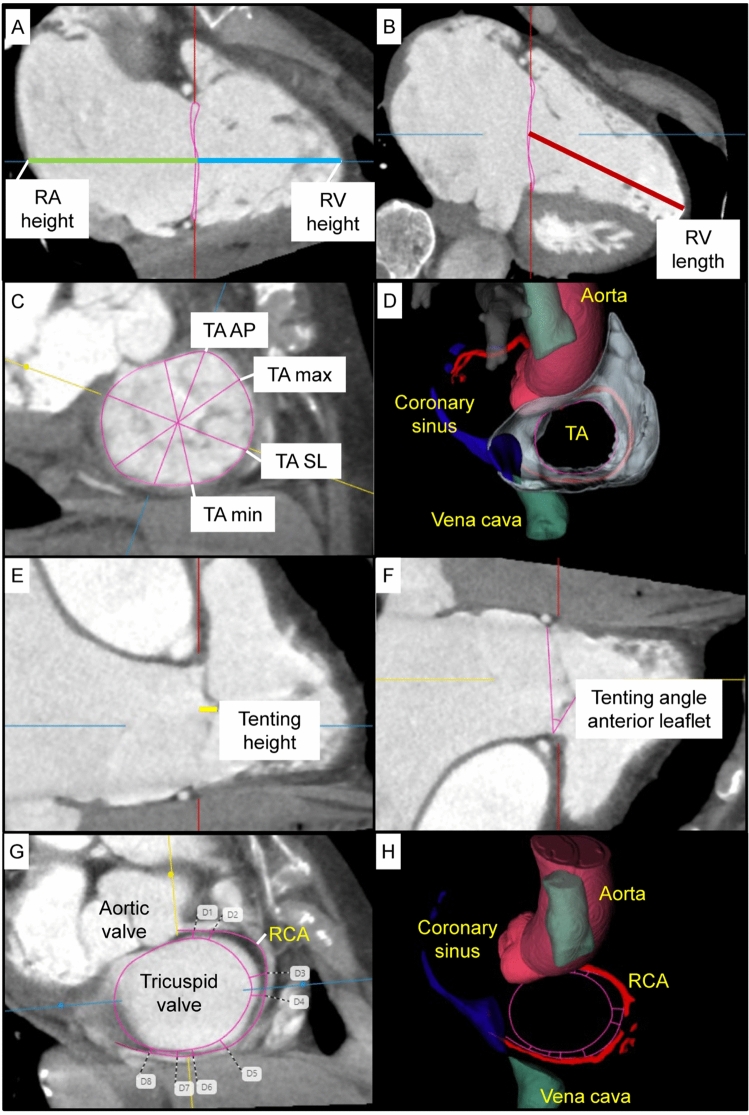
Computed tomography (CT) analysis of right atrial (RA) and right ventricular (RV) height (**A**), RV length (**B**), tricuspid annulus (TA) with antero-posterior (AP), septo-lateral (SL), minimal (min) and maximal (max) diameter (**C**, **D**), tenting height (**E**), tenting angle of the anterior leaflet (**F**), and distance between tricuspid annulus and right coronary artery (RCA, **G**, **H**).

CT parameters are presented in Table 2 and shown in Fig. 2. Right ventricular and atrial volumes were increased in the PA compared to the T-TEER group, but these differences did not reach statistical significance. This was consistent with a more pronounced tenting in PA patients, including the tenting angle of the septal leaflet, which was significantly larger in the PA compared to the T-TEER group. The tricuspid valve area and diameters did not differ significantly between the two groups. The distance between the tricuspid annulus and the RCA was larger in patients who underwent PA compared to T-TEER with a significant difference in segment D8 (Fig. 2). 35 patients in the PA (67%) and 23 patients in the T-TEER group (72%) had at least one segment with a distance to the RCA < 6 mm.

**Table 2:** Automated computed tomography analysis.

	T-TEER				PA				*p* Value for comparison of T-TEER and PA
	Entire cohort (*n* = 32)	Post-interventional TR grade ≤ 1 (*n* = 14)	Post-interventional TR grade > 1 (*n* = 18)	*p* Value for comparison of TR grade ≤ 1 versus > 1	Entire cohort (*n* = 52)	Post-interventional TR grade ≤ 1 (*n* = 19)	Post-interventional TR grade > 1 (*n* = 33)	*p* Value for comparison of TR grade ≤ 1 versus > 1	
LVEF, % ± SD	58 ± 11	56 ± 10	59 ± 11	0.382	59 ± 11	57 ± 13	60 ± 10	0.350	0.673
RA volume, ml ± SD	291 ± 96	296 ± 77	287 ± 111	0.807	307 ± 98	287 ± 69	320 ± 111	0.197	0.448
RA diameter (four-chamber view), mm ± SD	70 ± 10	71 ± 8	70 ± 11	0.743	70 ± 9	68 ± 9	71 ± 10	0.223	0.896
RA height, mm ± SD	75 ± 11	76 ± 11	74 ± 11	0.607	77 ± 11	76 ± 9	77 ± 12	0.606	0.388
RVEF, % ± SD	55 ± 8	56 ± 10	55 ± 7	0.912	55 ± 8	54 ± 9	55 ± 8	0.714	0.755
RVSV, ml (IQR)	125 (100–150)	113 (104–149)	136 (97–154)	0.411	134 (112–162)	131 (97–149)	147 (117–169)	0.078	0.246
RV EDV, ml (IQR)	224 (183–293)	209 (169–294)	236 (185–315)	0.428	248 (199–297)	208 (172–263)	272 (229–310)	0.079	0.570
RV ESV, ml (IQR)	97 (66–140)	95 (63–144)	98 (72–149)	0.511	110 (77–139)	99 (75–112)	114 (87–146)	0.108	0.470
RV height, mm ± SD	61 ± 13	61 ± 13	61 ± 13	0.950	64 ± 10	65 ± 7	64 ± 11	0.812	0.164
RV length, mm (IQR)	87 (76–97)	84 (73–94)	87 (79–101)	0.254	85 (80–91)	84 (78–91)	86 (80–91)	0.436	0.941
TV area mid-diastolic, mm^2^ (IQR)	1798 (1550–2049)	1720 (1594–2151)	1832 (1492–2005)	0.732	1701 (1556–2013), *n* = 50	1589 (1394–1921), *n* = 18	1720 (1657–2037), *n* = 32	0.150	0.683
TV perimeter mid-diastolic, mm ± SD	151 ± 18	151 ± 16	151 ± 21	0.946	149 ± 15, *n* = 50	147 ± 16, *n* = 18	151 ± 15, *n* = 32	0.391	0.613
SL TV diameter mid-diastolic, mm ± SD	48 ± 7	49 ± 6	48 ± 7	0.668	49 ± 5, *n* = 50	48 ± 6, *n* = 18	49 ± 5, *n* = 32	0.503	0.861
AP TV diameter mid-diastolic, mm (IQR)	47 (43–52)	48 (43–51)	47 (41–53)	0.933	45 (43–51), *n* = 50	45 (41–48), *n* = 18	45 (43–51), *n* = 32	0.337	0.353
Tenting height, mm (IQR)	8 (5–11)	6 (4–8)	9 (8–12)	0.004	10 (7–12)	9 (6–11)	11 (8–13)	0.131	0.098
Tenting volume, ml (IQR)	3 (1–5), *n* = 28	2 (1–3), *n* = 10	4 (1–9)	0.150	3 (1–6), *n* = 50	2 (1–4)	4 (2–7), *n* = 31	0.099	0.715
Tenting angle septal leaflet, ° (IQR)	17 (10–26)	16 (8–20)	24 (15–27)	0.033	27 (16–38), *n* = 51	22 (14–34)	29 (19–41), *n* = 32	0.070	0.010
Tenting angle anterior leaflet, ° (IQR)	23 (16–34)	18 (9–30)	26 (22–35)	0.023	26 (19–33), *n* = 51	25 (18–32)	27 (19–36), *n* = 32	0.370	0.304
Tenting angle posterior leaflet, (IQR)	23 (14–31)	19 (8–28)	26 (19–38)	0.071	26 (17–36), *n* = 51	22 (17–32)	27 (16–40), *n* = 32	0.320	0.362
Mean distance TA to RCA (D1-8), mm ± SD	8.4 ± 2.6	8.5 ± 2.5	8.4 ± 2.7	0.884	9.1 ± 2.2	8.9 ± 2.1	9.1 ± 2.2	0.754	0.231
Mean number of segments with proximity to the RCA* per patient, *n* (IQR)	3 (0–4)	3 (0–4)	3 (0–4)	0.969	2 (0–3)	2 (0–3)	1 (0–3)	0.751	0.148

*Proximity to the RCA was defined as a distance < 6 mm.

Continuous variables are shown as mean ± standard deviation (SD, normally distributed) or median with interquartile ranges (IQR, not normally distributed). T-TEER, Tricuspid transcatheter edge-to-edge repair; PA, Percutaneous annuloplasty; LVEF, Left ventricular ejection fraction; RA, Right atrial; RVEF, Right ventricular ejection fraction; RVSV, Right ventricular stroke volume; RV EDV, Right ventricular end-diastolic volume; RV ESV, Right ventricular end-systolic volume; RV height, Right ventricular height (corresponds to the length of the RV measured perpendicular to the tricuspid valve plane); RV length, Right ventricular length (corresponds to the length of the RV from apex to base); TV, Tricuspid valve; SL, Septolateral; AP, Anteroposterior; TA, Tricuspid annulus; RCA, Right coronary artery.

**Fig. 3:**
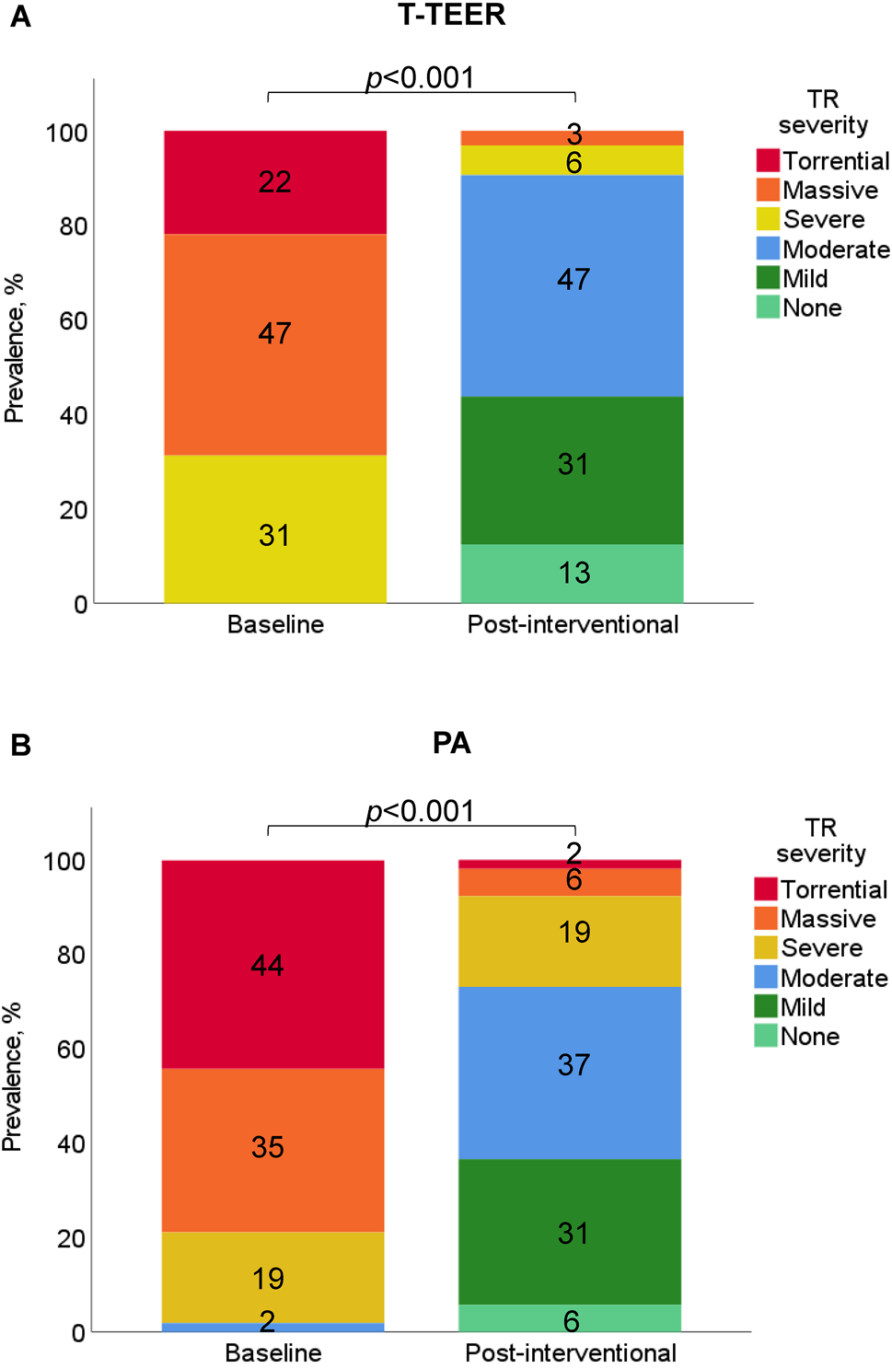
Severity of tricuspid regurgitation (TR) before and immediately after (**A**) tricuspid transcatheter edge-to-edge repair (T-TEER) or (**B**) percutaneous annuloplasty (PA).

Technical success was achieved in 100% of patients. After interventional therapy, PA and T-TEER patients showed a comparable improvement of TR severity (PA: 2.3 ± 0.8 vs. T-TEER: 2.3 ± 0.9, *p* = 0.688). Detailed TR grades before and after intervention are shown in Fig. 3. For further analysis, we divided both treatment cohorts into patients with a TR grade ≤ 1 and > 1 after intervention. We observed that patients with a post-interventional TR grade ≤ 1 had smaller right ventricular volumes and lower tenting heights, volumes, and angles regardless of PA or T-TEER treatment. This difference reached significance only for tenting height and angles. A comparison of patients with a TR improvement of ≥ 3 grades and < 3 grades showed similar results, except for the right atrial diameter in the two-chamber view in the PA group (TR improvement of ≥ 3 grades: 102 (95–109) mm vs. TR improvement < 3 grades: 92 (83–100) mm, *p* = 0.019).

### Predictors of post-interventional TR reduction

In the T-TEER group, the tenting height and the tenting angle of the anterior leaflet predicted a post-interventional TR grade ≤ 1 (tenting height: odds ratio [OR] 0.707 [95% confidence interval [CI] 0.537–0.931]; tenting angle: OR 0.919 [95% CI 0.850–0.993]). Neither right atrial volume, right ventricular end-systolic and end-diastolic volume, RVEF, tenting angle of the septal and posterior leaflet, tenting volume nor end-systolic and mid-diastolic tricuspid valve area as well as a more circular shape of the tricuspid annulus or the sphericity index predicted a TR grade ≤ 1 after T-TEER. Similarly, none of the mentioned parameters predicted a TR grade ≤ 1 after PA.

**Fig. 4:**
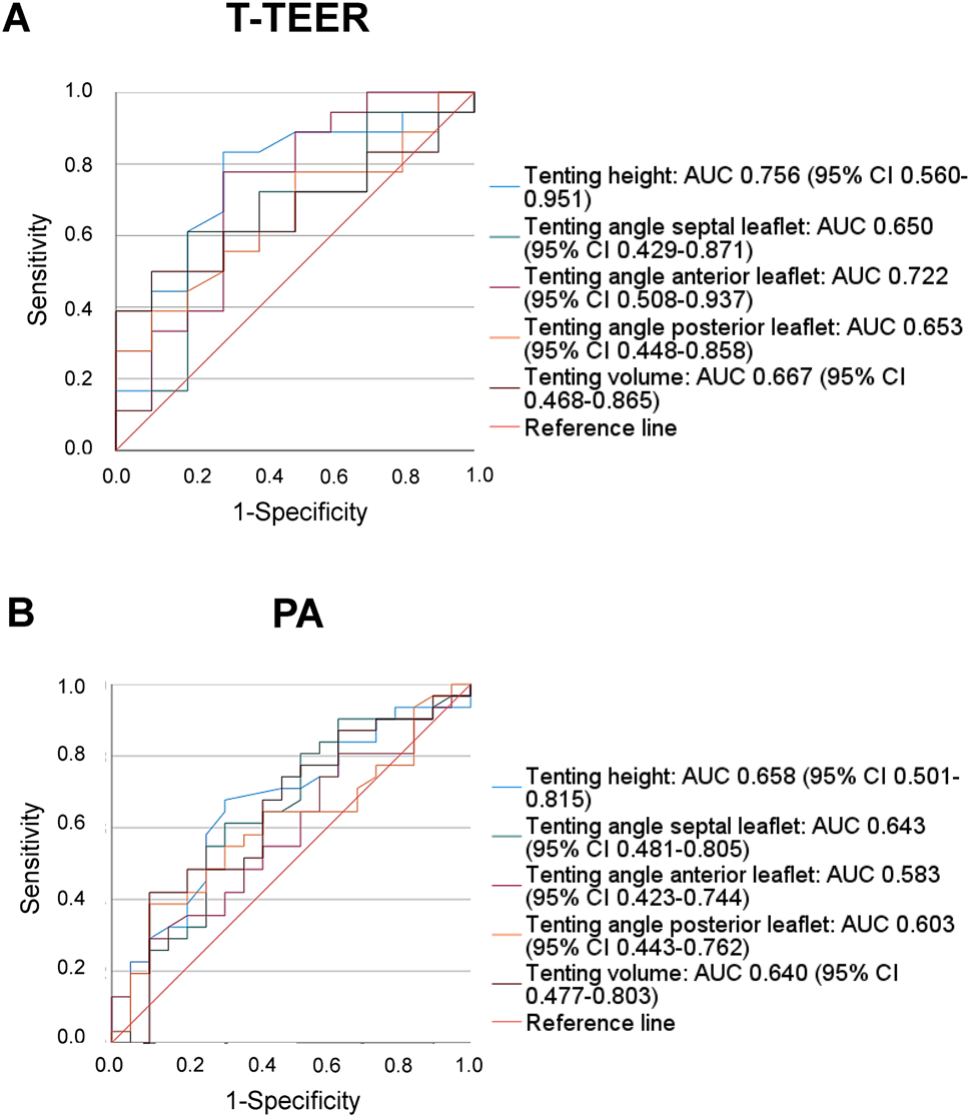
Receiver operating characteristic (ROC) analysis with area under the curve (AUC) and confidence interval (CI) of tenting height, tenting angle of the septal, anterior, and posterior leaflet and tenting volume. Patients after tricuspid transcatheter edge-to-edge repair (T-TEER) are presented in (**A**), patients after percutaneous annuloplasty (PA) in (**B**).

**Fig. 5:**
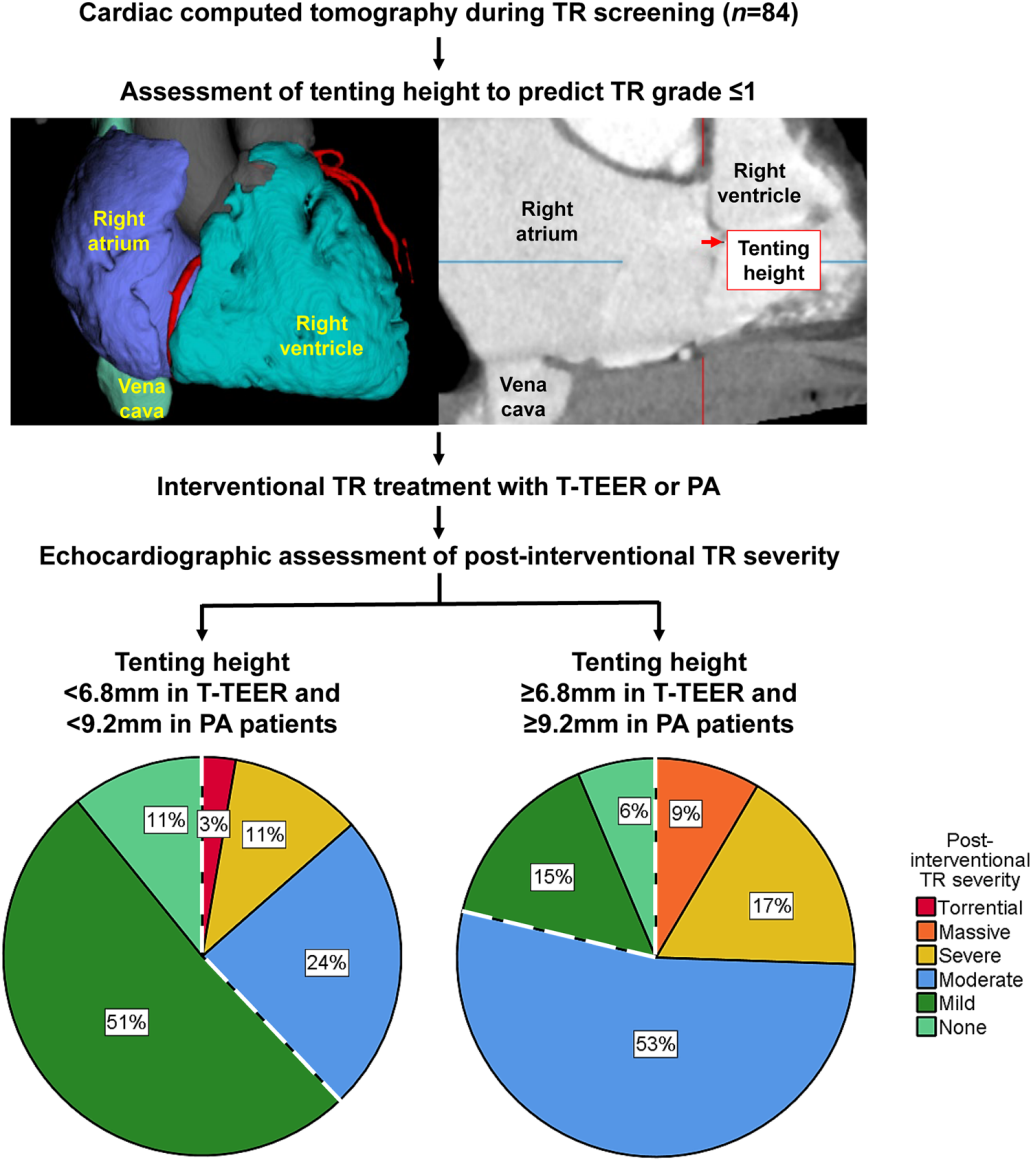
Tenting hight assessed by computed tomography (CT) was associated with a higher risk of residual TR after tricuspid transcatheter edge-to-edge repair (T-TEER) and percutaneous annuloplasty (PA). In ROC analysis, the threshold of 6.8 mm tenting height corresponded to a sensitivity of 83% and specificity of 70% in the T-TEER group and the threshold of 9.2 mm to a sensitivity and specificity of 68% in the PA group to determine TR grade > 1 post-interventional.

Furthermore, we conducted a ROC analysis with tenting height, volume, and angles to predict the outcome TR grade > 1 after intervention (Fig. 4). The tenting height showed the best diagnostic accuracy among evaluated parameters with an AUC of 0.756 (95% CI 0.560–0.951) in the T-TEER and 0.658 (95% CI 0.501–0.815) in the PA group. To determine post-interventional TR grade > 1, the threshold of 6.8 mm tenting height corresponded to a sensitivity of 83% and specificity of 70% in the T-TEER group, while the cut-off value of 9.2 mm was associated with a sensitivity and specificity of 68% in the PA group (Fig. 5).

### Correlation of CT and echocardiographic measurements

CT and echocardiographic measurements showed a significant correlation regarding left ventricular ejection fraction (LVEF, Pearson 0.574, *p* < 0.001, *n* = 84), right atrial diameter in the four-chamber view (Pearson 0.619, *p* < 0.001, *n* = 84), right ventricular length (Spearman-Rho 0.648, *p* < 0.001, *n* = 83) and tenting height (Pearson 0.603, *p* < 0.001, *n* = 84). Echocardiographic parameters are listed in Table 3.

**Table 3:** Echocardiographic measurements.

	T-TEER (*n* = 32)	PA (*n* = 52)	*p* Value
LVEF, % ± SD	51 ± 8	53 ± 11	0.339
RVD basal, mm ± SD	50 ± 10	49 ± 8	0.642
RVD length, mm ± SD	68 ± 13, *n* = 31	69 ± 10	0.694
RAD, mm ± SD	55 ± 10	55 ± 12	0.970
TAPSE, mm ± SD	18 ± 4, *n* = 30	18 ± 6, *n* = 51	0.608
RV FAC, % ± SD	45 ± 11, *n* = 28	41 ± 9, *n* = 51	0.175
Tenting height, mm ± SD	3 ± 3	6 ± 4	< 0.001
Gap ATL STL, mm (IQR)	4 (2–6), *n* = 28	5 (3–6), *n* = 37	0.288
Gap PTL STL, mm ± SD	5 ± 2, *n* = 29	5 ± 3, *n* = 37	0.695

Continuous variables are shown as mean ± standard deviation (SD, normally distributed) or median with interquartile ranges (IQR, not normally distributed). T-TEER, Tricuspid transcatheter edge-to-edge repair; PA, Percutaneous annuloplasty; LVEF, Left ventricular ejection fraction; RVD, Right ventricular diameter; RAD, Right atrial diameter in the four-chamber view; TAPSE, Tricuspid annular plane systolic excursion; RV FAC, Right ventricular fractional area change; ATL, Anterior tricuspid leaflet; STL, Septal tricuspid leaflet; PTL, Posterior tricuspid leaflet.

### Long-term follow-up

**Fig. 6:**
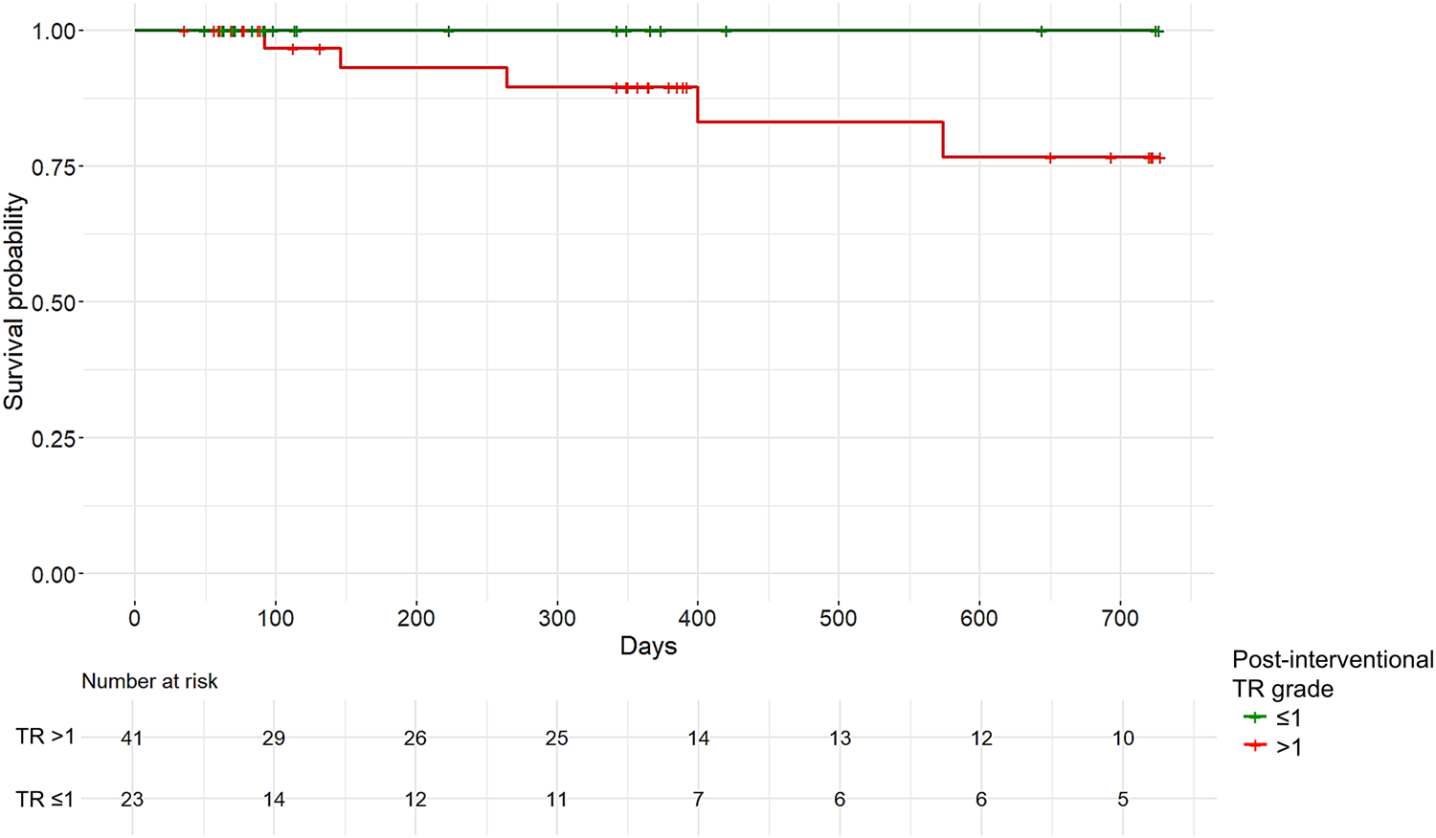
Kaplan–Meier curves of survival probability of patients with a post-interventional TR grade > 1 and ≤ 1 during a follow-up of 24 months. *TR* Tricuspid regurgitation.

The mean follow-up duration of 41 patients with a post-interventional TR grade > 1 was 370 ± 265 days, and that of 23 patients with a post-interventional TR grade ≤ 1 was 331 ± 300 days. Among the first group, five patients (12%) died during the follow-up, including two due to cardiovascular reasons. One patient (4%) with a post-interventional TR grade ≤ 1 died due to non-cardiovascular reasons 1046 days after intervention (*p* = 0.124 for intergroup comparison, Fig. 6).

## Discussion

The present study compared right heart morphology and function assessed by cardiac CT of patients who underwent T-TEER or PA and evaluated predictors of TR reduction after interventional therapy using automated deep learning CT algorithms. The principal findings include:

(i) PA patients presented larger right heart volumes and greater tenting. Nevertheless, PA and T-TEER resulted in a comparable TR improvement of, on average, 2–3 grades.
(ii) Expectedly (since safe distance to the RCA is a prerequisite for PA), the distance between the tricuspid annulus and the RCA was larger in the PA compared to the T-TEER group. However, 67% of patients who underwent PA had at least one segment which showed proximity to the RCA (defined as a distance < 6 mm).
(iii) Patients with a post-interventional TR grade ≤ 1 had lower tenting heights and angles. In the T-TEER group, the tenting height and angle of the anterior leaflet were associated with a post-interventional TR grade ≤ 1. We identified a tenting height threshold of 6.8 mm for T-TEER and 9.2 mm for PA to determine a post-interventional TR grade > 1.
(iv) Patients with a post-interventional TR grade > 1 showed a numerically higher mortality (12%) compared to patients with a TR grade ≤ 1 (4%).

### Right heart anatomy and function

In the present study, automated deep learning CT assessment was used to analyze right heart morphology and function. Measured median tricuspid valve area was slightly smaller compared to previous studies. Ingraham et al. reported an area of 19.5 ± 4.6 cm^2^ in patients who underwent surgical or interventional TR therapy and an area of 21.2 ± 2.25 cm^2^ in TR patients with medical treatment^23^. In the current study, patients with a residual TR grade > 1 exhibited slightly more enlarged right heart chambers compared to those with a TR grade ≤ 1. This is in line with results from mitral edge-to-edge repair (M-TEER). After the results of the COAPT and the MITRA-FR study, M-TEER is recommended for patients with left ventricular end-systolic diameter ≤ 70 mm according to the European Society of Cardiology (ESC) guideline on valvular heart disease (class IIa)^24,25^. Another relevant anatomical aspect for device selection is the distance between RCA and tricuspid annulus^8^. In clinical practice, a distance of at least 6 mm is recommended for PA treatment. However, 67% of patients in the PA and 72% of patients in the T-TEER group had at least one segment with a distance < 6 mm to the RCA. In the PA group, two patients required coronary intervention due to RCA perforation (3.8%), which was slightly higher than the reported prevalence by Nickening et al.^26^. Nevertheless, none of the segments for the two patients were at a distance < 6 mm. Therefore, further studies should investigate the minimum distance required and whether other risk factors contribute to perforation.

In addition to morphology, right ventricular function is an important outcome predictor in patients who underwent T-TEER or PA^11^. Patients referred for surgical or interventional TR therapy exhibited better right ventricular function in comparison to those undergoing medical treatment^23^. In the present study, mean RVEF assessed by CT and mean tricuspid annular plane systolic excursion (TAPSE) as well as right ventricular fractional area change (RV FAC) measured by echocardiography were in a normal range in T-TEER and PA patients at baseline.

### Tenting in TR

A significant tenting predominantly occurs in advanced and ventricular TR, but not in atrial TR as described by Schlotter et al.^17^. Consistent with this, patients with more pronounced tenting showed larger right ventricular end-diastolic volumes in the present study. Ingraham et al. observed an increased tenting height among patients undergoing medical management in contrast to those referred for surgical or interventional TR therapy^23^. CT measurements of mean tenting height were comparable to our values^23^. However, patients with a post-interventional TR > 1 exhibited similar (T-TEER) and slightly larger (PA) tenting heights compared to medically managed patients^23^. Nevertheless, a successful interventional treatment was reached despite elevated tenting heights in the current study. A TR reduction of at least one grade was achieved in all patients. In the T-TEER group, up to three devices were implanted in patients with a post-interventional TR grade ≤ 1 (one device: 7%, two devices: 71%, three devices: 21%) and up to two devices in patients with a post-interventional TR grade > 1 (one device: 22%, two devices: 78%, *p* = 0.080). In M-TEER, tenting is a known risk factor for single leaflet device attachments (SLDA)^27^. Two patients of our cohort (6%) presented with SLDA between the posterior and septal leaflet a few days after T-TEER. In both cases, the tenting heights formed by the posterior and septal leaflets were 0 and 6 mm, respectively. Thus, a causal relationship with detachment a few days after T-TEER remains unclear.

Moreover, more pronounced tenting was associated with TR grade > 1 after intervention in our study. This result is in line with findings from surgical annuloplasty in TR^28^. Tenting volume measured by 3D echocardiography was a predictor of residual TR after surgery^28^. In contrast, in the present study, tenting volume measured by CT had a lower AUC compared to tenting height in ROC analysis.

An alternative approach for patients with a significant tenting is orthotopic valve implantation^23^. The maximal mid-diastolic annulus diameter in T-TEER patients with a postinterventional TR grade > 1 was 52 (48–56) mm and in PA patients 51 (49–55) mm. As an example, the EVOQUE® valve (Edwards Lifesciences, Irvine, CA, USA) is available up to a diameter of 52 mm which reflects the unmet need for larger devices^23^. Nevertheless, besides technical advancements, TR patients should potentially be treated at earlier stages of the disease to prevent significant right ventricular enlargement and progression of tenting^23^. Further relevant predictors of residual TR comprised the leaflet-to-annulus index and the positioning of leads from CIEDs after T-TEER^29,30^.

In comparison to echocardiography, we observed larger values of tenting height measured by CT, although both measurements showed a correlation. One possible explanation might be the varying visibility of the leaflets in both modalities and the use of different phases as well as imaging perspectives with visualization of different leaflets. CT measurements were conducted at end-systolic phase using a two-chamber view, while echocardiographic parameters were evaluated in a late systolic phase in a four-chamber view. Tenting height measured by echocardiography also predicted a post-interventional TR grade ≤ 1 after T-TEER (OR 0.736 [95% CI 0.546–0.993]) but not after PA (OR 0.972 [95% CI 0.854–1.108]). The calculated AUC of echocardiographic tenting height was lower compared to CT analysis in both groups (T-TEER AUC 0.720 [95% CI 0.538–0.902] and PA AUC 0.510 [95% CI 0.352–0.669]).

### Limitations

Our study is limited by its single-center observational design. Results should be interpreted as hypothesis-generating due to limited case numbers. In a few patients, the measurement of tenting height using CT was challenging due to inadequate visualization of the leaflets. Furthermore, diameters of the right heart and tricuspid annulus are volume load dependent, which may lead to different measurements at screening (echocardiography and CT) and intervention. In addition to methodological limitations, both PA and T-TEER address different mechanism and were therefore used in patients with different anatomical characteristics such as atrial and ventricular TR. This must be taken into account when interpreting the results.

## Conclusion

Automated CT assessment of the right heart morphology and function as well as the tricuspid valve has the potential to guide decision making before interventional therapy of TR but needs confirmation in prospective studies. Significant tenting may identify patients with a high risk for residual TR after T-TEER and PA.

## Data Availability

The data that support the findings of this study are available from the corresponding author upon reasonable request.

## Abbreviations

ASE: American Society of Echocardiography
CIED: Cardiac implantable electronic device
CT: Computed tomography
EACVI: European Association of Cardiovascular Imaging
ESC: European Society of Cardiology
PA: Percutaneous annuloplasty
RCA: Right coronary artery
ROC: Receiver operating characteristic
TR: Tricuspid regurgitation
T-TEER: Tricuspid transcatheter edge-to-edge repair
